# Multiresistant Bacteria Isolated from Activated Sludge in Austria

**DOI:** 10.3390/ijerph15030479

**Published:** 2018-03-09

**Authors:** Herbert Galler, Gebhard Feierl, Christian Petternel, Franz F. Reinthaler, Doris Haas, Juliana Habib, Clemens Kittinger, Josefa Luxner, Gernot Zarfel

**Affiliations:** 1 Institute of Hygiene, Microbiology and Environmental Medicine, Medical University of Graz, 8010 Graz, Austria; he.galler@medunigraz.at (H.G.); gebhard.feierl@medunigraz.at (G.F.); franz.reinthaler@medunigraz.at (F.F.R.); doris.haas@medunigraz.at (D.H.); juliana.habib@medunigraz.at (J.H.); clemens.kittinger@medunigraz.at (C.K.); josefa.luxner@medunigraz.at (J.L.); 2 Institute of Laboratory Diagnostics and Microbiology, Klinikum-Klagenfurt am Wörthersee, 9020 Klagenfurt, Austria; christian.petternel@kabeg.at

**Keywords:** ESBL, MRSA, VRE, sewage sludge, PER-1

## Abstract

Wastewater contains different kinds of contaminants, including antibiotics and bacterial isolates with human-generated antibiotic resistances. In industrialized countries most of the wastewater is processed in wastewater treatment plants which do not only include commercial wastewater, but also wastewater from hospitals. Three multiresistant pathogens—extended spectrum β-lactamase (ESBL)-harbouring Enterobacteriaceae (Gram negative bacilli), methicillin resistant *Staphylococcus aureus* (MRSA) and vancomycin resistant Enterococci (VRE)—were chosen for screening in a state of the art wastewater treatment plant in Austria. Over an investigation period of six months all three multiresistant pathogens could be isolated from activated sludge. ESBL was the most common resistance mechanism, which was found in different species of Enterobacteriaceae, and in one *Aeromonas* spp. Sequencing of ESBL genes revealed the dominance of genes encoding members of CTX-M β-lactamases family and a gene encoding for PER-1 ESBL was detected for the first time in Austria. MRSA and VRE could be isolated sporadically, including one EMRSA-15 isolate. Whereas ESBL is well documented as a surface water contaminant, reports of MRSA and VRE are rare. The results of this study show that these three multiresistant phenotypes were present in activated sludge, as well as species and genes which were not reported before in the region. The ESBL-harbouring Gram negative bacilli were most common.

## 1. Introduction

Antibiotics in the environment represent a growing concern as their presence can promote the selection of antibiotic resistant bacteria (ARB) that pose a serious public health threat. ARB can further spread resistance genes in the environment by the mechanism of horizontal gene transfer through which environmental bacteria can then mediate pathogens to acquire antibiotic resistance genes [1,2,3,4]. Among the various sources accounting for the spread of ARB, organic wastes, including wastes of municipal and agricultural origin, have been widely reported to be potent reservoirs of ARB- harbouring genes for multidrug resistance. Previous studies have pointed out that numerous ARB and resistant genes have been detected in sewage sludge from municipal wastewater treatment plants (WWTPs) [4,5]. One predominant antibiotic resistance mechanism is the presence of Extended Spectrum β-Lactamases (ESBLs). ESBLs are of great microbiological and clinical importance in Enterobacteriaceae, especially *Escherichia coli* and *Klebsiella* spp. and other non-fermenting bacteria such as *Acinetobacter* spp. and *Pseudomonas aeruginosa* [6,7]. The presence of ESBL in surface water has been frequently demonstrated all over the world, which leads to the conclusion that if the bacteria in the water are able to host ESBL genes, then there will be ESBL in the population [8,9,10,11]. The spread of ESBL is enhanced by the localization of most of the ESBL genes on mobile genetic elements which allow the transmission of resistance genes to strains and species which are better adapted to the surface water environment. As a consequence of this, environmental bacteria can acquire resistance genes from e.g., strains of clinical origin [8,9,10,11]. Methicillin resistant *Staphylococcus aureus* (MRSA) originates from the clinical setting, as hospital acquired (HA)-MRSA. Nevertheless, MRSA strains started to spread among the healthy human population (so called community acquired CA-MRSA) and livestock (LA-MRSA) within the last decades like ESBL [12,13,14]. MRSA detections from environmental reservoirs, including surface water, are very rare compared to multiresistant Gram negative bacteria isolation. Although the population of Staphylococci flushed into the wastewater is high, the survival of Staphylococci in water environment seems to be much lower than that of Gram negative bacilli. Therefore reports of MRSA from this reservoir are mainly restricted to areas of high human influence, e.g., hospital waste water effluent [15,16,17]. Vancomycin resistant Enterococci (VRE) are one of the first documented antibiotic resistant bacteria with primary origin in animal farming. The rise of VRE was caused by the use of the glycopeptide avoparcin as a growth promoter from 1975 on. Although glycopeptide use was banned in livestock production in the European Union (1996) VRE are still present in animals and can also be found in hospital settings [18,19,20]. Hence VRE are present in waste and surface water, it seems that they are detected mostly sporadically. Furthermore, the number of studies covering this topic is limited. The aim of the present study was to investigate the presence of multidrug resistant bacteria such as ESBL-producing Enterobacteriaceae, MRSA and VRE in activated sludge in the second largest commercial WWTP in Austria.

## 2. Materials and Methods

### 2.1. Sample Collection

Activated sludge samples were collected in the period between September 2011 and February 2012, twice a month (except January) from the basin of the incoming untreated waste water at a sewage treatment plant (>500,000 population equivalent, wastewater load 1200 L/min) at the area of Graz, Styria/Austria. Wastewater entry into this treatment plant contained mainly domestic waste water and wastewater from hospitals in the area. The sludge samples were collected using sterile wide-mouth bottles. They were transported to the laboratory in a cooling box, where they were immediately stored in a refrigerator at 4–8 °C until processing within 24 h. In total, eleven sludge samples were collected in six measuring series.

### 2.2. Strain Isolation and Identification

Sludge samples were homogenized by vortexing for two minutes. For qualitative analysis, an amount of 1 mL from the homogenized sludge sample was suspended in 9 mL sterile saline solution (0.9% NaCl). In order to reduce the bacterial concentration, a decimal dilution series with saline solution was prepared.

ESBL isolation: 0.1 mL of each homogenized sludge sample was plated on chromID™ ESBL Agar (bioMérieux, Marcy-l’Etoile, France) and incubated for 24 h at 37 °C. Following incubation, ESBL positive colonies were determined based on the colour reaction of the ESBL-media (according to the manufacturer’s protocol). Additionally 0.1 mL of the sludge samples was incubated (24 h, 37 °C) in thioglycolate nutrient broth for enrichment, then 10 µL of the material was inoculated on ESBL-media and incubated for 24 h at 37 °C [21].

MRSA isolation: 0.1 mL of the homogenized solutions were plated on oxacillin agar (OXOID Ltd., Basingstoke, UK) and incubated for 48 h at 37 °C. Following incubation, MRSA positive colonies were determined based on the colour reaction of the OXA-media. Blue colonies were presumed to be MRSA.

VRE isolation: For selective enrichment of VRE, an amount of 1 mL from the homogenized sludge sample was inoculated in 9 mL BBL™ Enterococcosel™ broth (BD, Sparks, MD, USA) containing 6 mg/L Vancomycin. Enterococci growing in the media turn the colour of the media from light brown to dark brown or black. In order to reduce the bacterial concentration, a decimal dilution series with saline solution was prepared. Subsequently, 0.1 mL from each of the homogenized solutions were plated on chromID™ VRE Agar (bioMérieux, Marcy-l’Etoile, France) and incubated for 24 h at 37 °C. VRE positive colonies were determined based on the colour reaction of the VRE-media (according to the manufacturer’s protocol).

To obtain pure cultures, colonies growing on selective-media were transferred to blood agar (24 h, 37 °C). Identification was done using the Vitek^®^ MS (bioMérieux, Marcy-l’Etoile, France), an automated microbial identification system using matrix-assisted laser desorption/ionization time-of-flight mass spectrometry (MALDI-TOF-MS) and the biochemical-based VITEK^®^2 system (bioMérieux, Marcy-l’Etoile, France).

#### 2.2.1. Characterisation of ESBL Harbouring Gram Negative Bacilli

Identified Enterobacteriaceae were characterized for their resistance pattern by susceptibility testing according to EUCAST (EUCAST V2.0, 2012) [22], with ampicillin (AM), amoxicillin/clavulanic acid (AMC), piperacillin/tazobactam (TZP), cefalexin (CN), cefuroxime (CXM), cefoxitin (FOX), cefotaxime (CTX), ceftazidime (CAZ), cefepime (FEP), imipenem (IPM), meropenem (MEM), gentamicin (GM), trimethoprim/sulfamethoxazole (SXT), nalidixic acid (NA), ciprofloxacin (CIP), moxifloxacin (MOX), tetracycline (TE) and chloramphenicol (C) BD BBL^TM^ Sensi-Disc^TM^ paper discs (BD, Sparks, MD, USA). The inhibition zone diameters were interpreted according to EUCAST guidelines, except Enterobacteriaceae tested for tetracycline, chloramphenicol and nalidixic acid, which were evaluated by the Clinical Laboratory Standards Institute (CLSI, 2011) guidelines [23]. There are no interpretation guidelines for zone diameters of these three antibiotics according to EUCAST.

*E. coli* 25299 was used as reference. The inhibition zone diameters were interpreted according to EUCAST guidelines. The antimicrobials tested and resistance breakpoints applied can be found in the Supplementary Materials (Table S1). 

All isolates were screened for ESBL gene families, *bla*_CTX-M-1group_, *bla*_CTX-M-2group_, *bla*_CTX-M-9group_*, bla*_GES_*, bla*_SHV_, *bla*_TEM_, and *bla*_VEB_ by PCR and sequencing as described previously [24,25]. False-positive (not estimated Enterobacteriaceae) strains growing on ESBL-media with a green or brownish colour were identified as Pseudomonadales and Aeromonadales; we decided to include them in the study and therefore these strains were also screened for ESBL genes. Identified Pseudomonadales were characterized for their resistance pattern by susceptibility testing according to EUCAST (EUCAST V2.0, 2012); piperacilin/tazobactam (TZP), ceftazidime (CAZ), cefepime (FEP), meropenem (MEM), imipenem (IPM), amikacin (AN), gentamicin (GM), tobramycin (NN), ciprofloxacin (CIP) and levofloxacin (LEV).

#### 2.2.2. Determination of VRE

After isolation and identification of suspected VRE colonies antibiotic susceptibility was determined for ampicillin (AM), vancomycin (VA), teicoplanin (TEC), linezolid (LZD), tigecycline (TGC) and trimethoprim/sulfamethoxazole (SXT) by disc diffusion test according to the EUCAST guidelines (EUCAST V2.0, 2012). *E. faecalis* DSM20478 was used as reference. The minimal inhibition concentration (MIC) for 22 antibiotics was assigned by VITEK^®^2 using the AST-P586 card (bioMérieux, Marcy-l’Etoile, France). Resistance to the glycopeptides vancomycin and teicoplanin was confirmed by Etest (bioMérieux, Marcy-l’Etoile, France) according to the manufacturer’s instructions. The detection of the vancomycin resistance genes (*vanA/vanB)* was performed by real time PCR applying the Light cycler VRE Detection Kit (Roche, Branchburg, NJ, USA).

#### 2.2.3. Determination of MRSA

MRSA isolates were characterized for their resistance pattern by susceptibility testing according to EUCAST (EUCAST V2.0, 2012), tested with penicillin (P), cefoxitin (FOX), tetracycline (TE), erythromycin (E), clindamycin (CL), norfloxacin (NOR), amicazin (AN), gentamicin (GM), trimethoprim/sulfamethoxazole (SXT), fusidic acid (FA), rifampicin (RIF), linezolid (LZD) and mupirocin (MUP) using BD BBL^TM^ Sensi-Disc^TM^ paper discs (BD, Sparks, MD, USA). *Staphylococcus aureus* DSM799 was used as reference. PCR amplification was used to determine SCC*mec* type and presence of the Panton-Valentine-Leukozidin (PVL)-gene [26,27]. *Spa* typing was performed as described previously [28].

## 3. Results

All eleven investigated sludge samples revealed at least one kind of the screened multiresistant bacteria. In detail, ten of the eleven samples were positive for ESBL-harbouring Enterobacteriacea (82%), three samples were positive for MRSA (27%) and four samples for VRE (36%).

### 3.1. ESBL Gram Negative Bacilli Isolates

In total, 117 Enterobacteriaceae were screened for multidrug resistance phenotypically. Genetic analysis revealed 32 different positive isolates consisting of 21 *E. coli*, seven *Klebsiella pneumoniae*, three *Enterobacter* sp. and one *Raoultella ornithinolytica* (Table 1). Members of the CTX-M gene family were the most predominant ESBL genes.

The most detected ESBL gene was *bla*_CTX-M-15_, which was present in twelve (28.6%) of the 32 isolates followed by *bla*_CTX-M-1_, which was found in six (14.3%) isolates. In addition, five (11.9%) of the isolates harboured the *bla*_CTX-M-14_, three (7.1%) *bla*_CTX-M-3_, and one (2.4%) the *bla*_CTX-M-38_ gene. The non-CTX-M ESBL genes *bla*_SHV-2_ and *bla*_SHV-12_ were detected in four isolates from activated sludge.

Bacteria with ESBL phenotypes frequently carry additional antibiotic resistances. For the purpose of phenotypic differentiation, all ESBL *E. coli* isolates were tested for their susceptibility to 19 antibiotics. The antibiotic resistances of each of the investigated isolates are listed in Table 1.

No ESBL-producing Enterobacteriaceae showed resistance to tigecycline, amikacin and the carbapenems imipenem and meropenem. Penicillin-inhibitor combinations such as amoxicillin/clavulanic acid (53.1%, 17 of 32) and piperacillin/tazobactam (9.4%, 3 of 32) showed reduced efficacy against the ESBL producing Enterobacteriaceae. The cephamycin cefoxitin revealed resistance to eight (25%) isolates.

The most common co-resistance rates among the ESBL producing Enterobacteriaceae isolates to non-beta lactam antibiotics were detected for the quinolones, nalidixic acid 75% (24 of 32), ciprofloxacin 56.3% (18 of 32) and moxifloxacin 53.1% (17 of 32). The co-resistance for tetracycline was as high as 53.1% (17 of 32) and for the drug combination trimethoprim/sulfamethoxazole 50% (16 of 32). Co-resistance rates to aminoglycoside compounds were low with 34.4% (11 of 32) for gentamicin and 0% for amikacin.

Two ESBL-producing isolates were resistant to three antibiotics and 26 of the isolates were resistant to more than three antibiotic classes, which lead to a number of 28 ESBL isolates that could be assigned as multidrug resistant (Table 1).

Additional 25 Pseudomonadales were isolated from the ESBL screening plates but genetic analysing showed no positive confirmation for ESBL genes. Only one *Aeromonas* spp. isolate was tested positive for the ESBL gene *bla*_PER-1_. This isolate revealed resistance to ceftazidime and meropenem.

### 3.2. MRSA

Three MRSA isolates from three different activated sludge samples were detected. All three isolates harboured the mecA gen but were tested negative for PVL. Spa typing revealed one t032, with resistance to erythromycin, norfloxacin and gentamycin and one t067 with resistance to erythromycin, clindamycin and norfloxacin. The third MRSA with spa type t6613 was susceptible to all tested non beta-lactam antibiotics (Table 1).

### 3.3. VRE

VRE could be detected in four of eleven (36%) activated sludge samples represented by one Enterococcus isolate each. All four isolates were identified as *Enterococcus faecium* and harboured the *vanA* gene. All isolates showed highly similar resistance patterns. They were all resistant to ampicillin, teicoplanin, and vancomycin; three isolates showed additional resistance to trimethoprim/sulfamethoxazole (Table 1).

## 4. Discussion

The omnipresence of ESBL in environmental population of Enterobacteriaceae is widely demonstrated. The findings of this study go in full concordance with prior results. This includes also the isolated species (mostly *E. coli*) and the detected genes (CTX-M family) being dominant [8,11,29,30].

Other studies concerning *E. coli* from sewage sludge also reported tetracycline, ampicillin/clavulanic acid and trimethoprim/sulfamethoxazole as antibiotics with the highest non- susceptibility rate. These antibiotics showed the highest non-susceptibility in ESBL *E. coli* from Austrian sewage sludge as well [31,32]. Regarding co-resistance, the isolates did not show a reduced occurrence as can be observed in ESBL isolates from surface waters, without direct wastewater influence. Resistance to quinolones was very common and most of the isolates could be classified as multiresistant (resistance to three or more tested antibiotic classes). Environment and residence time in the WWTP seem not to favour a potential adaptation process in the ESBL population. The permanent entry of ARB from different sources in the activated sludge basin and the horizontal gene transfer are the dominant factors for the composition of resistant bacteria. Selection pressure due to different substances, does not seem to have enough time in this environment to contribute to resistance development [9,10,30,33,34,35,36,37]. Therefore, the isolates of this study reflect rather the situation of clinical ESBL isolates where this kind of co-resistance and multiresistance is dominant. Interestingly the majority of the ESBL Enterobacteriaceae isolates remained susceptible to the tested 4th generation cephalosporin (cefepim).

The isolation of a PER-1 producing *Aeromonas* spp. is more remarkable. There are reports of PER-1 based ESBL (also in *Aeromonas*) in European surface waters, nevertheless clinical isolates with this enzyme are reported rarely. In Austria, this is the first PER-1 producer documented so far [38,39].

The MRSA isolates from the sewage sludge can be linked to hospital settings. A multiresistant phenotype including the aminoglycoside gentamicin is a typical characteristic of hospital acquired (HA)-MRSA. T032 is a common *spa* type of the ST22-MRSA-IV (Barnim epidemic MRSA strain). It is the most prevalent HA-MRSA in Europe and has spread in Austria since the beginning of this decade. The second gentamicin resistant (t067) isolate can be linked to the so called paediatric clone. The resistance pattern of this MRSA isolate, with the exotic *spa* type t6613, showed similarity with CA-MRSA, but did not harbour the genes for the PVL toxin [40,41].

In general MRSA isolates from surface water are rather rare, with only low number of analysed isolates. Therefore an estimation which of the three MRSA types is more dominant in water environment is difficult to make [15,16,42,43].

VRE isolates showed nearly identical features in terms of species, gene and resistance pattern. Likewise MRSA, VRE isolates were only investigated and isolated in few studies compared to studies with ESBL isolates. This is remarkable because in contrast to Staphylococci, Enterococci have a much better ability to survive in surface water and they are indicator bacteria for water quality assessment [44,45,46]. Therefore, the exclusivity of *vanA* isolates is more likely to be based on the low number of sludge isolates. Furthermore other environmental VRE isolates from Austria revealed also *vanB* [44,45,46,47,48,49].

However, there is much evidence that confirms the presence of diverse and plentiful ARB in fertilizer produced from livestock animals [50,51]. There appears to be significant variability on wastewater management across different industrialized countries. In high income countries sewer connectivity is generally high, whereas in many middle and low income countries sewer connectivity is low and untreated sewage is discharged mainly to surface water bodies [52,53].

## 5. Conclusions

Wastewater treatment plants serve as a collection basin of multiresistant bacteria. In the investigated activated sludge samples all three screened multiresistant phenotypes were present, with ESBL harbouring Gram negative bacilli representing the most common ones. The study shows for the first time in Austria, the presence of VRE in WWTP and the first detection of a PER-1 mediated ESBL. All these multiresistant bacteria have the potential to spread in other ecological niches and therefore further monitoring and measures for reduction should be taken into consideration.

## Figures and Tables

**Table 1:** Detected resistance genes and resistance pattern of all isolates. Non *E. coli* Enterobacteriaceae are automatically set resistant to AM according EUCAST.

Isolate ID	Sample	Date	Species	Resistance Genes	Resistance Pattern ^a^
ESBL-01	KS1	2011-09	*E. coli*	*bla_CTX-M-15_, bla_TEM-1_* ^b^	AM, AMC, CN, CXM, FOX, CTX, GM, SXT, CIP, MXF, CAZ, FEP, TE, NA
ESBL-02	KS5	2011-10	*E. coli*	*bla_CTX-M-15_*	AM, AMC, CN, CXM, CTX, CIP, MXF, CAZ, FEP, TE, NA
ESBL-03	KS5	2011-10	*E. coli*	*bla_CTX-M-1_, bla_TEM-1_*	AM, CN, CXM, CTX, GM, SXT, TE, C
ESBL-04	KS5	2011-10	*E. coli*	*bla_TEM-1_*	AM, CN, CXM, CTX, SXT, CIP, CAZ, FEP, TE, NA, C
ESBL-05	KS5	2011-10	*E. coli*	*bla_CTX-M-1_*	AM, AMC, CN, CXM, CTX, SXT, CIP, MXF, NA, C
ESBL-06	KS5	2011-10	*E. coli*	*bla_CTX-M-1_*	AM, CN, CXM, CTX, FEP
ESBL-07	KS6	2011-11	*E. coli*	*bla_CTX-M-15_*	AM, CN, CXM, CTX, SXT, CIP, MXF, CAZ, FEP, TE, NA, C
ESBL-08	KS6	2011-11	*E. coli*	*bla_CTX-M-3_*	AM, AMC, CN, CXM, CTX, SXT, CIP, MXF, FEP, TE, NA, C
ESBL-09	KS6	2011-11	*E. coli*	*bla_CTX-M-3_, bla_TEM-1_*	AM, AMC, CN, CXM, FOX, CTX
ESBL-10	KS7	2011-12	*E. coli*	*bla_CTX-M-14_, bla_TEM-1_*	AM, CN, CXM, CTX, CIP, MXF, NA
ESBL-11	KS7	2011-12	*E. coli*	*bla_CTX-M-3_, bla_TEM-1_*	AM, CN, CXM, CTX, FEP, NA
ESBL-12	KS7	2011-12	*E. coli*	*bla_CTX-M-1_*	AM, CN, CXM, CTX, GM, SXT, CIP, MXF, CAZ, FEP, TE, NA
ESBL-13	KS7	2011-12	*E. coli*	*bla_CTX-M-15_, bla_TEM-1_*	AM, CN, CXM, CTX, FEP
ESBL-14	KS7	2011-12	*E. coli*	*bla_CTX-M-1_, bla_TEM-1_*	AM, CN, CXM, CTX, SXT, FEP, TE
ESBL-15	KS7	2011-12	*E. coli*	*bla_CTX-M-14_*	AM, AMC, CN, CXM, CTX, TE, NA
ESBL-16	KS7	2011-12	*E. coli*	*bla_CTX-M-14_, bla_TEM-1_*	AM, AMC, CN, CXM, CTX, TE, NA
ESBL-17	KS8	2011-12	*E. coli*	*bla_CTX-M-14_, bla_TEM-1_*	AM, AMC, CN, CXM, CTX, CIP, MXF, CAZ, TE, NA
ESBL-18	KS9	2012-01	*E. coli*	*bla_CTX-M-38_*	AM, AMC, CN, CXM, FOX, CTX, GM, CIP, MXF, TZP, CAZ, FEP, TE, NA
ESBL-19	KS10	2012-02	*E. coli*	*bla_CTX-M-1_*	AM, CN, CXM, CTX, SXT, FEP, TE
ESBL-20	KS11	2012-04	*E. coli*	*bla_CTX-M-15_, bla_SHV-11_* ^b^*, bla_TEM-1_*	AM, CN, CXM, CTX, CAZ, FEP
ESBL-21	KS11	2012-04	*E. coli*	*bla_CTX-M-14_*	AM, CN, CXM, CTX, SXT, CIP, MXF, NA
ESBL-22	KS1	2011-09	*E. kobei*	*bla_SHV-2_*	AM, AMC, CN, CXM, FOX, CTX, GM, CAZ, FEP, NA, C
ESBL-23	KS4	2011-09	*E. kobei*	*bla_SHV-2_*	AM, AMC, CN, CXM, FOX, CTX, GM, CAZ, NA, C
ESBL-24	KS9	2012-01	*E. cloacae*	*bla_CTX-M-15_, bla_TEM-1_*	AM, AMC, CN, CXM, FOX, CTX, GM, SXT, CIP, MXF, TZP, CAZ, TE, NA
ESBL-25	KS2	2011-09	*K. pneumoniae*	*bla_CTX-M-14_, bla_SHV-2_*	AM, CN, CXM, CTX, GM, SXT, FEP
ESBL-26	KS6	2011-11	*K. pneumoniae*	*bla_SHV-12_*	AM, CN, CXM, CTX, CIP, MXF, CAZ, NA, C
ESBL-27	KS7	2011-12	*K. pneumoniae*	*bla_CTX-M-15_, bla_SHV-1_* ^b^ *, bla_TEM-1_*	AM, AMC, CN, CXM, CTX, GM, SXT, CIP, MXF, CAZ, TE, NA
ESBL-28	KS7	2011-12	*K. pneumoniae*	*bla_CTX-M-15_, bla_SHV-1_, bla_TEM-1_*	AM, AMC, CN, CXM, CTX, GM, SXT, CIP, MXF, CAZ, FEP, TE, NA
ESBL-29	KS8	2011-12	*K. pneumoniae*	*bla_CTX-M-15_, bla_SHV-11_*	AM, AMC, CN, CXM, FOX, CTX, SXT, CIP, MXF, CAZ, FEP, NA, C
ESBL-30	KS9	2012-01	*K. pneumoniae*	*bla_CTX-M-15_, bla_SHV-1_, bla_TEM-1_*	AM, CN, CXM, CTX, CIP, MXF, NA, TE
ESBL-31	KS11	2012-04	*K. pneumoniae*	*bla_CTX-M-15_, bla_SHV-11_*	AM, AMC, CN, CXM, CTX, GM, SXT, CIP, MXF, CAZ, TE, NA
ESBL-32	KS11	2012-04	*R. ornithinolytika*	*bla_SHV-2_*	AM, AMC, CN, CXM, FOX, CTX, TZP, CAZ, NA
ESBL-33	KS7	2011-12	*Aeromonas.* sp.	*bla_PER-1_*	CAZ, MEM
VRE-01	KS4	2001-09	*E. faecium*	*van*A	AM, TEC, VA, SXT
VRE-02	KS7	2011-12	*E. faecium*	*van*A	AM, TEC, VA, SXT
VRE-03	KS8	2011-12	*E. faecium*	*van*A	AM, TEC, VA, SXT
VRE-04	KS10	2012-02	*E. faecium*	*van*A	AM, TEC, VA
MRSA-01	KS3	2011-09	*S. aureus*	*mec*A	P, FOX, E, NOR, GM
MRSA-02	KS5	2011-10	*S. aureus*	*mec*A	P, FOX
MRSA-03	KS6	2011-11	*S. aureus*	*mec*A	P, FOX, E, CC, NOR, GM

^a^ AM, ampicillin; AMC, amoxicillin/clavulanic acid; TZP, piperacillin/tazobactam; CN, cephalexin; CXM, cefuroxime; FOX, cefoxitin; CTX, cefotaxime; CAZ, ceftazidime; FEP, cefepime; MEM, meropenem; CIP, ciprofloxacin; MXF, moxifloxacin; GM, gentamicin; SXT, trimethoprim/sulfamethoxazole; TE, tetracycline; NA, nalidixic acid; C, chloramphenicol; TEC, teicoplanin; VA, vancomycin; P, penicillin; E, erythromycin; NOR, norfloxacin. ^b^ Resistance genes *bla*_TEM-1_, *bla*_SHV-1_ and *bla*_SHV-11_ encoding non-extended-spectrum-β-lactamases.

## Supplementary Materials

The following tables are available online at http://www.mdpi.com/1660-4601/15/3/479/s1, Table S1: Antibiotics, disk content and breakpoints used for disk susceptibility testing according to the EUCAST guidelines (EUCAST V2.0, 2012).
