# Supplementary material for: Multiresistant Bacteria Isolated from Activated Sludge in Austria
Source: Int J Environ Res Public Health. 2018 Mar 9;15(3):479. doi: 10.3390/ijerph15030479 (PMC5877024; doi:10.3390/ijerph15030479)
Supplement: Supplementary file 1 [file ijerph-15-00479-s001.pdf]

Table S1: Antibiotics, disk content and breakpoints used for disk susceptibility testing according to the EUCAST guidelines (EUCAST V2.0, 2012).

| Species | Antibiotic | Disk content (µg) | Zone diameter (mm) breakpoints[a] | |
|---|---|---|---|---|
| | | | S ≥ | R < |
| Enterobacteriaceae | ampicillin (AM) | 10 | 14 | 14 |
| | amoxicillin/clavulanic acid (AMC) | 20+10 | 17 | 17 |
| | cephalexin (CN) | 30 | 12 | 12 |
| | cefuroxime (CXM) | 30 | 18 | 18 |
| | cefoxitin (FOX) | 30 | 19 | 19 |
| | cefotaxime (CTX) | 5 | 20 | 17 |
| | moxifloxacin (MFX) | 5 | 20 | 17 |
| | ciprofloxacin (CIP) | 5 | 22 | 19 |
| | trimethoprim/sulfamethoxazole (SXT) | 1,25+23,75 | 16 | 13 |
| | gentamicin (GM) | 10 | 17 | 14 |
| | meropenem (MEM) | 10 | 22 | 16 |
| | imipenem (IPM) | 10 | 22 | 16 |
| | cefepime (FEP) | 30 | 24 | 21 |
| | ceftazidime (CAZ) | 10 | 22 | 19 |
| | piperacillin/tazobactam (TZP) | 30+6 | 20 | 17 |
| | tetracycline (TE)[b] | 30 | 15 | 11 |
| | chloramphenicol ( C)[b] | 30 | 18 | 12 |
| | nalidixic acid (NA)[b] | 30 | 19 | 13 |
| Enterococcus spp. | ampicillin (AM) | 2 | 10 | 8 |
| | vancomycin (VA) | 5 | 12 | 12 |
| | teicoplanin (TEC) | 30 | 16 | 16 |
| | linezolid (LZD) | 10 | 19 | 19 |
| | tigecycline (TGC) | 15 | 18 | 15 |
| Staphylococcus spp. | penicillin (P) | 1 | 26 | 26 |
| | cefoxitin (FOX) | 30 | 22 | 22 |
| | tetracycline (TE) | 30 | 22 | 19 |
| | erythromycin (E) | 15 | 21 | 18 |
| | clindamycin (CL) | 2 | 22 | 19 |
| | norfloxacin (NOR) | 10 | 17 | 17 |
| | amicazin (AN) | 30 | 18 | 16 |
| | gentamicin (GM) | 10 | 18 | 18 |
| | trimethoprim/sulfamethoxazole (SXT) | 1,25+23,75 | 17 | 14 |
| | fusidic acid (FA) | 10 | 24 | 24 |
| | rifampicin (RIF) | 5 | 26 | 23 |
| | linezolid (LZD) | 10 | 19 | 19 |
| | mupirocin (MUP) | 200 | 30 | 18 |
| Pseudomonas spp. | piperacillin/tazobactam (TZP) | 30+6 | 19 | 19 |
| | ceftazidime (CAZ) | 10 | 16 | 16 |
| | cefepime (FEP) | 30 | 18 | 18 |
| | meropenem (MEM) | 10 | 24 | 18 |
| | imipenem (IPM) | 10 | 20 | 17 |
| | amicazin (AN) | 30 | 18 | 15 |
| | gentamicin (GM) | 10 | 15 | 15 |
| | tobramycin (NN) | 10 | 16 | 16 |
| | ciprofloxacin (CIP) | 5 | 25 | 22 |
| | levofloxacin (LEV) | 5 | 20 | 17 |

[a] Zone diameter value used to indicate susceptible (S) and resistant (R)

[b] Enterobacteriaceae tested for tetracycline, chloramphenicol and nalidixic acid were evaluated in conformity with Clinical Laboratory Standards Institute (CLSI, 2011) guidelines. There are no interpretation guidelines for zone diameters of these three antibiotics according to EUCAST.
